# Oral Myiasis: A Structured Review of Risk Factors, Clinical Presentation, and Treatment

**DOI:** 10.7759/cureus.116137

**Published:** 2026-09-12

**Authors:** Nicolás Monsalves, Felipe Estrada, Jose Venegas

**Affiliations:** 1 Dentistry, Universidad de Los Andes, Santiago, CHL; 2 Dentistry, Universidad del Desarrollo, Santiago, CHL; 3 Oral and Maxillofacial Surgery, Universidad de Chile, Santiago, CHL

**Keywords:** cochliomyia hominivorax, dipteran larvae, ivermectin, neglected tropical disease, oral myiasis, parasitic infestation, risk factors

## Abstract

Oral myiasis is an uncommon parasitic infestation caused by dipteran larvae that invade living or necrotic oral tissues, and it is associated with poor oral hygiene, periodontal disease, neurological or psychiatric disability, malignancy, and social vulnerability. This structured review aimed to characterize the risk factors, clinical presentation, and therapeutic alternatives for strictly oral myiasis. A literature search was conducted in PubMed/MEDLINE, SciELO, and Scopus to identify case reports and case series involving humans with disease restricted to the oral cavity, published in Spanish or English between 2000 and 2026. To focus on contemporary evidence, only cases published from 2020 onward were included. Fourteen primary studies were analyzed. There was a slight male predominance, and the patients ranged from a five-month-old infant to a 91-year-old adult, with most presenting a neurological, psychiatric, or oncological predisposing condition. The most frequently affected sites were the anterior maxilla and the palate. In most reports, the larva was not identified to the species level; when identified, the agents were Chrysomya bezziana, Musca domestica, and Sarcophaga ruficornis. Management was consistently multimodal, combining mechanical removal of larvae with systemic antibiotics, antiparasitic therapy (ivermectin), surgical debridement, and topical agents. Oral myiasis mainly affects medically and socially vulnerable patients and can be life-threatening in critically ill hosts; its management requires a multidisciplinary approach, and awareness among healthcare professionals is essential for early diagnosis and reducing associated morbidity.

## Introduction and background

Oral myiasis is an uncommon parasitic infestation in humans, characterized by the invasion of living or necrotic oral tissues by dipteran larvae, mainly belonging to the families Calliphoridae, Sarcophagidae, and Oestridae. These larvae feed on living tissue, necrotic tissue, or body fluids and can cause significant tissue destruction, pain, inflammation, bleeding, and, in severe cases, mutilation of the oral and maxillofacial tissues [1]. Although oral myiasis has classically been documented in case reports and case series, recent systematic reviews have synthesized this scattered body of evidence. A 2025 systematic review that screened more than 12,000 records and included 95 publications concluded that the condition predominantly occurs in regions with unfavorable socioeconomic conditions and poor hygiene, and that the treatment of choice consists of mechanical removal of larvae, surgical debridement, and oral ivermectin [2]. Similarly, a systematic review of human myiasis in sub-Saharan Africa confirmed its association with poverty, a warm climate, and limited access to healthcare [3].

Several predisposing factors are recognized. These include poor oral hygiene, periodontal disease, labial incompetence, ulcerated lesions, open wounds, and advanced oral neoplasms (for example, squamous cell carcinoma), as well as systemic conditions such as immunosuppression, neurological or mental disability, and situations of social vulnerability [4,5]. The disease is more prevalent in tropical and subtropical regions, mainly affects males and individuals with social or medical risk factors, and can occur both in local populations and in travelers. Species distribution and clinical presentation vary by region, with Cochliomyia hominivorax (C. hominivorax), Dermatobia hominis, and Oestrus ovis being the most relevant agents depending on the geographic context [6,7].

From an epidemiological standpoint, available data confirm that human myiasis affects both residents and travelers and follows a marked social gradient, with a higher burden among men and populations with limited access to healthcare [6]. In the local context, C. hominivorax is the predominant etiological agent of human and animal myiasis in southern South America, including Chile, where its distribution is influenced by climatic and geographic factors and is concentrated in rural and peri-urban zones [8]. This regional relevance warrants a closer look at the oral presentation of the disease, which remains comparatively undercharacterized. Preventive measures operate on two axes: vector control (waste management, physical barriers, and insecticides in high-risk areas) and the reduction of individual risk factors, particularly the maintenance of oral hygiene and the timely management of oral wounds and lesions in vulnerable patients. In the hospital setting, surveillance of dependent patients and infection-control protocols help prevent nosocomial cases [9,10,11].

The most frequent treatment, in agreement with the international and regional literature, is based on the combination of mechanical removal of larvae and the use of ivermectin, orally or subcutaneously, according to the severity and extent of the condition [12,13]. Manual extraction of larvae with forceps must be performed carefully to avoid rupturing them, which could increase local inflammation and hinder resolution [14,15]. In extensive or difficult-to-access cases, suction aspiration can be a useful complementary technique, especially in complex periocular or facial areas [16]. The use of topical agents such as ether or creolin can facilitate extraction by inducing the migration of larvae toward the surface; subsequently, surgical debridement of necrotic tissue, when necessary, contributes to infection control and healing [17].

Although several reviews have addressed human and oral myiasis, most either pool oral presentations with extraoral or cutaneous presentations or focus on isolated aspects such as etiology or a single treatment. The present review addresses this gap by applying an explicit, reproducible selection process to assemble a contemporary series of cases with involvement restricted to the oral cavity, and by integrating their risk factors, anatomical distribution, causative agents, and treatment in a single structured synthesis oriented to the dental and maxillofacial clinician. Accordingly, the aim of this structured review is to characterize the risk factors, clinical presentation, and therapeutic alternatives for strictly oral myiasis.

## Review

Materials and methods

A structured literature review was conducted to characterize the risk factors, clinical presentation, and therapeutic alternatives for oral myiasis. A search was performed in the PubMed/MEDLINE, SciELO, and Scopus databases. The search strategy combined free-text terms and descriptors using the Boolean operators AND and OR to identify all reported cases of oral myiasis, including "oral myiasis," "oral cavity myiasis," "gingival myiasis," "maxillofacial myiasis," "oral larval infestation," and "palatal myiasis" (Table 1). Language filters (Spanish and English) and publication-date filters (2000-2026) were applied. The final search was performed in September 2026. The reference lists of the selected articles and recent reviews were also screened manually. This review did not include a formal risk-of-bias appraisal or a registered protocol; the included reports were synthesized qualitatively.

**Table 1:** Search strategy by database Language filters: Spanish and English. Publication date: 2000-2026. Last search: September 2026

Database	Search strategy	Records
PubMed/MEDLINE	("oral myiasis" OR "oral cavity myiasis" OR "gingival myiasis" OR "maxillofacial myiasis" OR "oral larval infestation" OR "palatal myiasis")	485
SciELO	(oral myiasis) OR (gingival myiasis) OR (maxillofacial myiasis) OR (miasis oral) OR (miasis gingival)	22
Scopus	TITLE-ABS-KEY("oral myiasis" OR "oral cavity myiasis" OR "gingival myiasis" OR "maxillofacial myiasis" OR "oral larval infestation" OR "palatal myiasis")	167
Total		674

The inclusion criteria were case reports and case series involving humans; articles published in English or Spanish; publications from 2000 to 2025; and infestation by dipteran larvae with involvement restricted to the oral cavity proper. For the purposes of this review, "oral" involvement was operationally defined as infestation of intraoral structures, including the gingiva, alveolar ridges, hard and soft palate, buccal mucosa, labial mucosa (inner aspect of the lips), tongue, floor of the mouth, and tooth extraction sockets. Cases confined to the external (cutaneous) lip or perioral skin, as well as cases of maxillofacial or facial myiasis without clear intraoral involvement, were excluded. Studies conducted in animals, cases of generalized myiasis, and other non-human studies were also excluded. When a report described mixed involvement, it was included only if the intraoral component was clearly documented and the extracted data could be specifically attributed to the intraoral involvement.

Study identification and selection were documented using a Preferred Reporting Items for Systematic Reviews and Meta-Analyses (PRISMA)-style flow diagram (this is a structured literature review and not a formal systematic review). The database search identified a total of 674 records: 485 in PubMed/MEDLINE, 22 in SciELO, and 167 in Scopus. After 119 duplicate records were removed, 555 records were screened by title and abstract, of which 393 were excluded for not meeting the eligibility criteria, including records unrelated to the topic, veterinary or animal studies, non-oral forms of myiasis, and agricultural or entomological reports.

**Figure 1:**
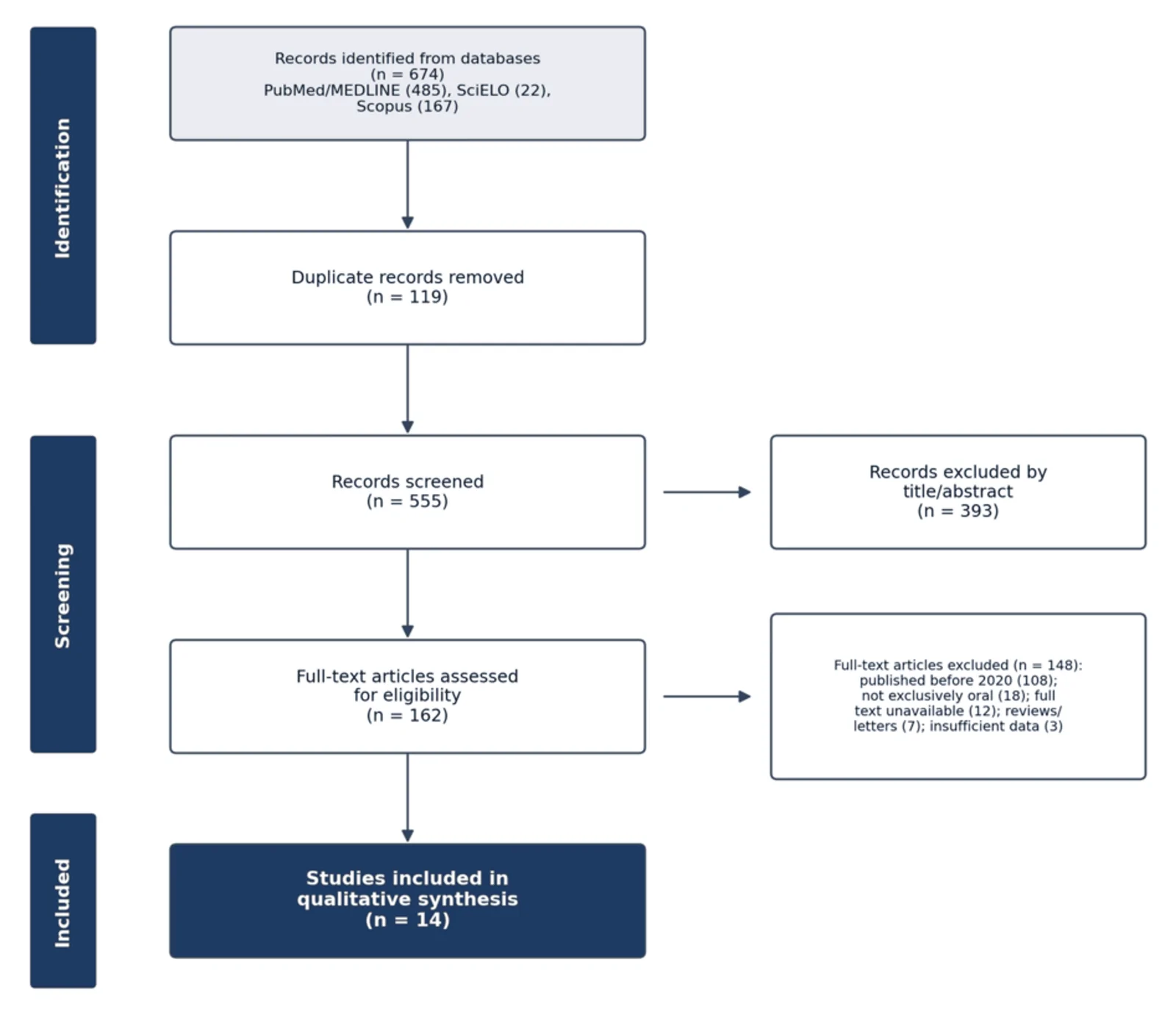
PRISMA flow diagram depicting the study selection The diagram summarizes the identification, screening, and inclusion of studies retrieved through the database search PRISMA: Preferred Reporting Items for Systematic Reviews and Meta-Analyses

The remaining 162 full-text articles were assessed for eligibility, and 148 were excluded for the following reasons: publication before 2020 and therefore outside the recency criterion (n = 108); involvement that was not exclusively oral (n = 18); full text not available (n = 12); reviews or letters rather than primary case reports (n = 7); and insufficient extractable clinical data (n = 3). Thus, 14 primary studies met all eligibility criteria and were included in the qualitative synthesis (Figure 1). Study selection and data extraction were performed independently by two reviewers, and disagreements were resolved by consensus. For each study, the following variables were extracted: country, sample size and patient demographics, affected anatomical site, clinical presentation, larval species, treatment, and comorbidities.

Results

Study Selection

The database search identified 674 records (485 in PubMed/MEDLINE, 22 in SciELO, and 167 in Scopus). After removing 119 duplicates, 555 records were screened by title and abstract, and 393 were excluded. Of the 162 full-text articles assessed for eligibility, 148 were excluded, chiefly because they were published before 2020 (n = 108, based on the recency criterion), were not exclusively oral (n = 18), had an inaccessible full text (n = 12), were reviews or letters (n = 7), or provided insufficient extractable data (n = 3). Fourteen primary studies met all eligibility criteria and were included in the qualitative synthesis (Figure 1); their main characteristics are summarized in Table 2 [18-31].

**Table 2:** Summary of the included primary studies of oral myiasis (2020-2026) "Not specified (Diptera)" indicates the original report did not identify the larva to the species level M: male; F: female; y: years; mo: months; IV: intravenous; VP: ventriculoperitoneal; COX1: cytochrome oxidase subunit 1; C.: Chrysomya; M.: Musca; S.: Sarcophaga

Study	Country	Sample	Affected site	Clinical presentation	Identified species/agent	Treatment	Comorbidity
Vasanthakumar et al., 2020 [18]	India	1 (M, 12 y)	Palatal gingiva, anterior maxilla	Larvae in palatal region (visual diagnosis); poor oral hygiene	Not specified (Diptera)	Mechanical removal with hemostat; oral ivermectin	Cerebral palsy, poor oral hygiene, mouth breathing, incompetent lips
Shenoi et al., 2020 (case 2) [19]	India	1 (F, 18 y)	Upper labial vestibule (anterior maxilla)	Pain and swelling of the anterior maxilla; diffuse erythematous cavitation; 73 maggots	Chrysomya bezziana (morphology-confirmed)	Turpentine oil; curettage; irrigation (saline, hydrogen peroxide, povidone-iodine, metronidazole); ceftriaxone	Congenital cerebral palsy
Astekar et al., 2020 [20]	India	1 (F, 25 y)	Anterior maxilla, canine to canine (labial and palatal)	Enormous quantity of larvae, fetid odour, bleeding gingiva, bone exposure	C. bezziana (epidemiological ID)	Turpentine oil; manual removal (>40 larvae); saline and povidone irrigation; amoxicillin-clavulanate and metronidazole IV; debridement	Mentally challenged, cerebral palsy, anterior open bite, lip incompetence
Gadda et al., 2021 [21]	India	1 (M, 62 y)	Anterior maxillary alveolus	Painful throbbing wound; necrotic ulceration, bleeding; palatal perforation; severe halitosis	Not specified (Diptera)	Topical turpentine oil; mechanical removal with curettage; debridement; oral penicillin; extraction	Hypertension, hemiplegia, diabetes; amlodipine-induced gingival hyperplasia; mouth breathing
Zhou et al., 2021 [22]	China	1 (infant, 5 mo)	Oral cavity (lips, oral mucosa) and one larva in ear canal	Lip swelling preventing mouth closure; oral ulcers and erosions; 10 maggots in mouth	Sarcophaga ruficornis (morphology and COX1)	Manual removal of maggots; supportive care (concurrent chemotherapy)	Acute myeloid leukemia
Coronel-Gamarra and Lezcano, 2022 [23]	Paraguay	1 (F, 91 y)	Maxillary residual roots (post-extraction wound)	80 larvae in post-extraction wound (dependent elderly)	Not specified (Diptera); facultative	Modified Dakin irrigation; mechanical removal (80 larvae); extraction of teeth 2.4-2.5; ivermectin (6 mg); amoxicillin	Elderly, hypertension, prior myocardial infarction, socioeconomic vulnerability, open-mouth sleeping
da Câmara et al., 2023 [24]	Brazil	1 (M, 55 y)	Dorsum of the tongue	Crateriform wound with numerous larvae; prior facial trauma	Not specified (Diptera)	Manual removal; ivermectin (15 mg); metamizole sodium; 0.12% chlorhexidine	Mentally challenged; psychiatric hospitalization
Sangalette et al., 2023 [25]	Brazil	1 (M, 82 y)	Bilateral maxillary vestibule and alveolar ridge	Dysphagia, local pain, excess salivation; extensive necrobiosis	Not specified (Diptera)	Oral ivermectin; topical ether; larval removal; surgical debridement; IV cephalothin	Stroke, terminal cancer, implant-supported fixed prosthesis
Monawwer et al., 2023 [26]	Pakistan	1 (F, 45 y)	Buccal mucosa and palate	Persistent oral bleeding; painful midface swelling; fungating mass; ulcerative lesion	Not specified (Diptera); invasive fungal co-infection	Larval removal; debridement; itraconazole; metronidazole; IV colistin	Scar epilepsy, hydrocephalus with VP shunt (post-meningoencephalitis)
Singh et al., 2023 [27]	India	1 (F, 67 y)	Floor of the mouth and anteroinferior gingivolabial sulcus	Pain, mild lower-lip swelling; inflamed gingiva, fetid odor, tooth mobility; deep cavities	Not specified (Diptera)	Turpentine oil; manual removal (~90 larvae); saline/povidone irrigation; ivermectin 6 mg; metronidazole	Metastatic ovarian carcinoma, immunosuppression (chemotherapy)
Shubham et al., 2024 [28]	India	1 (M, 45 y)	Inner aspect of upper and lower lips (labial mucosa)	Foul smell and blood from mouth; tissue discoloration; maggots; fever, altered sensorium	Not specified (Diptera)	Tooth extraction; manual removal; saline wash and turpentine-soaked gauze; IV antibiotics (ceftriaxone, vancomycin)	Immunocompetent; prior dental implant; admitted for fever and altered sensorium
Akrim and El Hakkouni, 2025 [29]	Morocco	1 (M, 21 y)	Oral cavity (periodontium, buccal/palatal mucosa)	Multiple larvae 10 days post-op; fetid odor, calculus, caries, periodontal pockets; fatal (septic shock)	Musca domestica	Manual extraction; metronidazole	Chronic smoking, ischemic stroke, glioblastoma (post-intubation)
Bahia et al., 2025 [30]	Brazil	1 (F, 16 y)	Hard and soft palate (multiple foci)	Oral lesion, fever; dyspnea from larval migration to oropharynx	Not specified (Diptera)	Manual removal; IV ceftriaxone, clindamycin, ivermectin; topical nitrofurazone; surgical debridement	Severe meningitis sequelae: spastic paralysis, epilepsy; bacterial pneumonia
Xiao et al., 2026 [31]	China	1 (M, 77 y)	Gingiva (teeth 15-17) and between lips and teeth	Gingival hyperemia and swelling, bleeding on probing; residual roots, deep caries; 20-30 maggots	Not specified (Diptera)	Manual removal (20-30 larvae); hydrogen peroxide and saline irrigation; soft-tissue debridement; anti-infective therapy; glycemic control	Type 2 diabetes, diabetic ketoacidosis coma, septic shock (post-intubation)

Patient Characteristics

The 14 included studies comprised 14 patients. There was a slight male predominance, with eight patients (57%) compared with six female patients (43%). The age range was notably broad, spanning from a five-month-old infant [22] to a 91-year-old adult [23], with patients represented across all age groups. Geographically, the cases originated predominantly from India (six cases) and Brazil (three cases), followed by China (two cases) and single reports from Paraguay, Pakistan, and Morocco [18-31]. A predisposing condition was identifiable in every case. Neurological or psychiatric conditions were the most frequent (present in nine patients), including cerebral palsy, epilepsy, stroke, and psychiatric hospitalization; malignancy was present in four patients, while diabetes and immunosuppression were present in two patients each. Poor oral hygiene and functional dependence were recurrent findings across the series.

Anatomical Sites

The infestation involved the anterior maxilla and maxillary structures in six cases and the palate (hard and/or soft) in five cases, supporting a predilection for the anterior and superior regions of the oral cavity. Other affected sites included the gingiva (three cases), the maxillary or labial vestibule (two cases), the buccal mucosa (two cases), as well as the labial mucosa, dorsum of the tongue, and floor of the mouth. Several cases were associated with tooth extraction sites, residual roots, or an implant-supported prosthesis, while a substantial proportion occurred in intubated or critically ill patients [25,29,31].

Etiological Agents

In most reports (10 of 14, 71%), the larva was not identified to the species level and was recorded only as belonging to the order Diptera. When species identification was performed, the agents were Chrysomya bezziana (two cases) [19,20], Musca domestica (one case) [29], and Sarcophaga ruficornis (one case) [22], the latter being confirmed through both morphological examination and molecular analysis of the cytochrome oxidase subunit 1 gene. This recurrent lack of species-level identification represents a consistent limitation of the primary literature.

Clinical Presentation

The clinical presentation was dominated by the local effects of larval invasion. The most frequently described features were the visible presence of moving larvae, tissue swelling, pain, fetid odor or halitosis, gingival bleeding, and ulcerated or necrotic lesions; deep cavitations, bone exposure, and palatal perforation were reported in advanced cases. Most infestations remained localized; a fatal outcome occurred in two critically ill patients in whom the underlying systemic condition, rather than the myiasis itself, was the primary determinant [29,31].

Treatment

Management was consistently multimodal. Mechanical removal of larvae was performed in virtually all cases (13 of 14) and constituted the therapeutic cornerstone. It was often combined with systemic antibiotics (10 cases), antiparasitic therapy with ivermectin, oral or topical (six cases), surgical debridement (six cases), and topical turpentine oil as an adjuvant to induce larval emergence (five cases). Tooth extraction of involved or residual teeth was required in four cases. This combination of mechanical, antiparasitic, and antiseptic measures, together with management of the underlying condition, achieved resolution of the infestation in the majority of patients.

Discussion

Oral myiasis is an uncommon infestation in humans, closely associated with precarious socioenvironmental conditions, debilitating diseases, and the absence of timely medical care. Although its global prevalence is low, it is reported more frequently in warm and humid climates, especially in developing countries. In this review, most cases originated from countries in South Asia and Latin America, with India and Brazil being the main countries of origin, followed by isolated reports from Colombia, Turkey, Pakistan, Morocco, and Indonesia. This distribution is consistent with the greater density of dipterans in tropical and subtropical climates and with the findings of recent systematic reviews, which identify low-income regions as bearing a disproportionate burden of the disease [2,3].

In most of the reviewed studies, the patients presented with risk factors such as alcoholism, systemic diseases, mental or motor disability, low socioeconomic status, or homelessness. Poor oral hygiene was an almost constant finding and was identified as a key predisposing factor for infestation [32]. The most frequent anatomical location was the anterior third of the oral cavity, especially the palate and maxilla. Labial incompetence, defined as the inability to completely close the lips at rest, was recognized as a relevant risk factor because it facilitates the continuous exposure of oral tissues to the external environment and increases the risk of larval colonization [15,33].

The demographic distribution of the reviewed cases ranged from a five-month-old infant to a 91-year-old adult, with a predominance of patients with some form of vulnerability. Two patterns of predisposition were apparent: on one hand, young and middle-aged adults with neurological or psychiatric conditions and limited capacity for self-care, and on the other, elderly or critically ill patients with functional dependence, malignancy, or metabolic decompensation. Although a male predominance has traditionally been reported, and was also observed here, women accounted for a substantial proportion of the cases. A recurring feature across nearly all cases, irrespective of age or sex, was the coexistence of poor oral hygiene with a condition that impairs the ability to maintain it, whether neurological, psychiatric, oncological, or socioeconomic in nature.

A phenomenon that deserves attention is nosocomial oral myiasis. The case of an intubated patient after glioblastoma surgery, in which Musca domestica larvae were identified in the oral cavity with a fatal outcome, illustrates the vulnerability of critically ill patients with a permanently open mouth and compromised hygiene, and underscores the need for vector-control protocols in the hospital environment [10,29]. Likewise, the association of oral myiasis with advanced squamous cell carcinoma, documented in several reports, underscores that exposed and necrotic tumor lesions constitute a favorable niche for larval colonization in patients with delayed access to oncological care [15,34].

The anatomical distribution observed further reinforces the role of local predisposing factors. The anterior maxilla, the palate, and the labial and gingival regions were the most frequently affected sites, a pattern consistent with their accessibility to ovipositing flies, particularly in patients with lip incompetence, anterior open bite, or mouth breathing. Several cases were directly related to dental procedures or oral rehabilitation, including infestations arising at recent extraction sockets, over residual roots, and even around an implant-supported fixed prosthesis. These observations underscore that any open oral wound, exposed necrotic tissue, or retained prosthesis in a vulnerable patient constitutes a potential nidus for larval colonization, and they highlight the importance of reinforcing postoperative oral hygiene and follow-up, particularly in patients who cannot maintain it independently.

The clinical presentation described across the reviewed reports typically included the visible presence of larvae, pain, swelling, halitosis, gingival bleeding, and ulcerated or necrotic lesions, with deep cavitation and bone exposure in advanced cases. Regarding the etiological agents, in most reports the larva was not identified to the species level; when species-level identification was performed, the agents were Chrysomya bezziana, Musca domestica, and Sarcophaga ruficornis, the latter being confirmed by molecular analysis in a neonatal case. This recurrent absence of species-level identification, often attributable to larval rupture during removal or the lack of entomological expertise, is a consistent limitation of the primary literature and limits any inference about species-specific behavior.

The most frequently reported treatment was mechanical removal of larvae, under local or general anesthesia, followed by irrigation with saline solution or chlorhexidine. Oral ivermectin (6-12 mg) was frequently used and showed utility in multiple or deep infestations [14,35]. Turpentine oil was used to induce the emergence of larvae in several reports, although the clinical evidence is limited and its safety in humans is not established; in vitro studies have demonstrated a larvicidal effect against Chrysomya megacephala, without assessing tissue safety, and its use has been questioned because of its potential toxicity [36,37]. Antibiotics, mainly metronidazole and doxycycline, were used to prevent or treat secondary bacterial infections; metronidazole also appeared useful in controlling the malodor of necrotic wounds, although the available evidence is of low quality [38].

Across the reviewed cases, the antiparasitic agent ivermectin emerged as a consistent pharmacological backbone, administered as single or repeated doses (commonly 6-12 mg) and particularly valuable in extensive infestations, in anatomically inaccessible sites, and in uncooperative patients for whom repeated mechanical removal is difficult. Its use, combined with mechanical extraction, was associated with favorable resolution in the majority of reports. Topical agents were also used to facilitate larval extraction, and their mechanism of action deserves clarification. Turpentine oil and ether act as volatile, irritant substances that create a hypoxic and noxious local environment, prompting the anchored larvae to migrate toward the surface, whereas creolin acts as a phenolic antiseptic with a similar irritant effect; in all cases, the aim is to induce larval emergence and facilitate subsequent mechanical removal.

However, the widespread use of turpentine oil in particular warrants caution: although it appears repeatedly in the literature and in the present cases, the supporting evidence is anecdotal, its safety on human mucosa has not been formally established, and in vitro larvicidal activity has been demonstrated without accompanying tissue-toxicity data. Systemic antibiotics, chiefly metronidazole and amoxicillin, were frequently added to control or prevent secondary bacterial infection, and in one case invasive fungal coinfection required antifungal therapy. These patterns suggest that, while a combined mechanical and ivermectin-based approach is supported by the available evidence, adjuvant topical agents should be reevaluated in favor of safer alternatives, and antimicrobial use should be guided by the clinical context rather than applied uniformly.

The occurrence of oral myiasis in contexts of social exclusion or functional decline should alert healthcare professionals to the importance of a comprehensive evaluation that not only addresses parasite removal but also the management of underlying conditions. A multidisciplinary approach, which combines physical measures (removal, hygiene, extraction), pharmacological measures (ivermectin, antibiotics), and social measures (follow-up, institutional support), is essential to prevent recurrences and major complications, including extension toward the paranasal sinuses, the skull base, or the cervical structures. Care for vulnerable populations requires enhanced training of health personnel to enable early diagnosis and timely management. Finally, climate change and globalization may favor the expansion of the species that cause myiasis toward new regions, making it necessary to strengthen surveillance systems and adapt preventive measures to emerging epidemiological scenarios.

This work has several limitations that should be acknowledged. First, as a structured review based on case reports and case series, the included evidence is inherently of low level and susceptible to publication bias; no formal risk-of-bias or quality appraisal of the individual studies was performed. Accordingly, the findings should be regarded as descriptive rather than confirmatory. Second, the small number of included studies (n = 14) and the marked heterogeneity of the therapeutic protocols preclude any quantitative synthesis or determination of the comparative effectiveness of the different treatments; the reported outcomes cannot establish the superiority of one approach over another.

Third, to focus on contemporary evidence, we restricted inclusion to cases published from 2020 onward, which necessarily excluded older reports and may limit the representativeness of the series. Fourth, the search was restricted to three databases and to studies in Spanish or English, and a proportion of full texts could not be retrieved; both factors introduce the possibility of availability and language bias. Finally, the frequent absence of species-level identification limits inferences about the specific larval agents involved. Despite these limitations, the structured synthesis of the recently published experience is useful for guiding the clinical recognition and management of this uncommon condition.

## Conclusions

Within the scope of the cases reviewed here, oral myiasis emerged as an uncommon condition occurring almost exclusively in patients with clear predisposing factors, chiefly poor oral hygiene combined with neurological or psychiatric disability, advanced periodontal disease, or functional dependence in older or terminally ill patients. In this series, the lesions were concentrated in the anterior maxilla, palate, and gingiva; the larvae were most often not identified to the species level and, when identified, corresponded mainly to Chrysomya bezziana and Musca species; and management consistently combined mechanical removal of larvae with oral ivermectin and, where indicated, antiseptic irrigation and systemic antibiotics. These observations, drawn directly from the included reports, support mechanical removal plus ivermectin as the therapeutic backbone and identify impaired self-care as the central modifiable risk factor.

Beyond these case-derived findings, and consistent with the broader literature, oral myiasis can be regarded as a largely preventable, neglected condition whose burden is shaped by socioeconomic and environmental determinants. From that wider perspective, prevention may benefit from health education, environmental and vector control, and surveillance of vulnerable and hospitalized patients, together with appropriate training for healthcare professionals to facilitate early diagnosis. These broader recommendations derive from external evidence rather than from the present cases and should be interpreted accordingly.
